# Use of Direct‐to‐Consumer Telemedicine: A Qualitative Study Exploring the Thoughts, Perspectives and Experiences of Australian Adults

**DOI:** 10.1111/hex.70847

**Published:** 2026-08-31

**Authors:** Andrew Turner, Kate Churruca, Samantha Spanos, Thomas Courtney‐Stubbs, Darran Foo, Louise A. Ellis

**Affiliations:** ^1^ Centre for Healthcare Resilience and Implementation Science, Australian Institute of Health Innovation Macquarie University Sydney New South Wales Australia; ^2^ Healthdirect Australia Limited Sydney New South Wales Australia; ^3^ Lifespan Health and Wellbeing Research Centre, School of Psychological Sciences Macquarie University Sydney New South Wales Australia

**Keywords:** direct‐to‐consumer, general practice, healthcare access, patient experience, telehealth, telemedicine

## Abstract

**Introduction:**

Following the COVID pandemic, direct‐to‐consumer (DTC) telemedicine services that offer medical expertise via remote electronic interfaces have surged in popularity. This paper explores the perspectives and experiences of patients using DTC telemedicine services in Australia, including their perceptions of benefits, concerns and interactions with traditional general practice.

**Methods:**

This was a qualitative study using semi‐structured interviews, which were recorded and transcribed. Thematic analysis was conducted using NVivo, with a combination of inductive and deductive coding. Participants were recruited from a previous survey on user experiences with up to five different DTC telemedicine services.

**Results:**

Thirteen participants had experience with DTC telemedicine services for women's health, skin care, men's health and women's fertility. Four themes were identified: (1) **Convenience, efficiency and greater access**—flexible appointment scheduling, reduced wait times and the online user interface were major benefits of DTC telemedicine services. Participants also highlighted the capacity of DTC telemedicine to enhance healthcare accessibility for those living rurally or with mobility challenges; (2) **The value of anonymity**—DTC telemedicine facilitated care for potentially stigmatised conditions such as obesity and erectile dysfunction, encouraging some patients to seek care they might otherwise avoid; (3) **Concerns about inappropriate use of medication**—participants questioned whether ease of access might lead to inappropriate prescribing, and (4) **Interactions with general practice**—DTC telemedicine was perceived as more of a supplement to general practice rather than a replacement. Despite this, participants indicated that negative experiences with GPs contributed to DTC telemedicine use.

**Conclusion:**

DTC telemedicine was viewed as a convenient and efficient alternative to general practice for certain health issues. However, concerns were identified regarding prescribing safety, continuity of care and inequity in access. If regulatory gaps are addressed, collaboration with GPs improved and disparities in access minimised, DTC telemedicine has the potential to play a complementary role in strengthening Australia's primary healthcare system.

**Patient and Public Involvement:**

This research was undertaken in partial fulfilment of the requirements of a Doctor of Medicine program. Given the timeframe allotted for undertaking this research, formal PPIE was not possible. However, two members of the research team had experience using the services discussed in this study and drew upon these experiences in developing interview questions and interpreting findings.

## 1 Introduction

The COVID‐19 pandemic fuelled the integration of telehealth into the mainstream of clinical service provision. In this context, direct‐to‐consumer (DTC) telemedicine services offering medical expertise via remote electronic interfaces have surged in popularity, particularly in Australia, despite its largely taxpayer‐funded healthcare system [1, 2, 3]. This rapid adoption has outpaced research into their effectiveness as healthcare services, resulting in a scarcity of evidence regarding patient outcomes, safety, as well as the implications of DTC telemedicine for traditional general practice in Australia.

DTC telemedicine providers vary in the composition of services offered, from those that offer ongoing treatment for specific health issues such as weight loss, skin concerns, hair loss and sexual health, to those that provide on‐demand medical services such as prescriptions, medical certificates or general medical advice [2, 3, 4]. Patients typically initiate the use of DTC telemedicine via websites or smartphone apps [4]. Upon selecting the desired service, they may then complete a medical questionnaire, send photos, participate in a clinical review with a licensed practitioner over the phone, have treatment delivered and provide updates on treatment progress [4, 5].

While telemedicine in general may not encompass all aspects of a face‐to‐face encounter, there appears to be a credible argument for its use in consultations that involve discussing test results, do not require physical examination, involve tracking treatment progress, or in circumstances where healthcare is otherwise difficult for patients to access due to remoteness or travel barriers [6, 7]. The proposed benefits of DTC telemedicine, specifically, include improved access for patients living in rural or remote areas, convenience, shorter wait times, reduced costs and increased autonomy [2, 7, 8, 9, 10, 11, 12]. Despite these benefits, DTC telemedicine has raised ethical and safety concerns, including inadequate regulation, uncertain treatment efficacy, lack of integration with general practice, deceptive advertising, overdiagnosis, privacy issues and the absence of physical examination when it may be indicated [2, 4, 5, 9, 11].

One study exploring the impacts of DTC telemedicine consultations on primary healthcare consumption in Stockholm, Sweden, showed that compared to a face‐to‐face consultation, the use of DTC telemedicine increased levels of subsequent primary healthcare consumption both within a month, and to a lesser extent over 2–6 months [13]. In Stockholm, DTC telemedicine is subsidised by the Swedish Government, so these results may not be generalisable to other healthcare systems like Australia's, where DTC telemedicine sits outside the taxpayer‐funded system, and patients are required to pay the full fee to access it.

Historically, Australians have largely accessed general practice without out‐of‐pocket costs through bulk billing, whereby general practitioners (GPs) are remunerated through the universal health insurance scheme, Medicare, and accept the Medicare rebate as full payment for the consultation. The decline in bulk‐billing rates from 89% in 2020–2021 to 78% in 2024 has increased the financial burden on patients seeking GP care, and may be contributing to the coinciding rise in alternative models such as DTC telemedicine in Australia [14]. This is compounded by a growing GP workforce shortage, with GP supply falling behind demand, driven by Australia's ageing demographic and increasing chronic disease burden [15]. This workforce shortage is especially pronounced in rural, regional and remote areas.

A new model of DTC telemedicine service has gained traction in Australia; one that is paid for privately on a subscription basis outside of the Medicare system, provides focused treatment for specific health issues (e.g., weight management, skin concerns), delivers medications to the patient's door and incorporates both synchronous and asynchronous interactions with allocated physicians employed by the service and with whom patients usually have no established doctor–patient relationship [2, 3, 4]. The rapid expansion of these services has occurred in a regulatory grey zone, with a clear shortage of research to guide policy [1]. Despite this, some policy changes and movements towards increased regulation have already been made. For example, the Therapeutic Goods Administration (TGA) has banned compounded GLP‐1 receptor agonists, while the Medical Board of Australia has revised its telehealth guidelines to discourage prescribing through questionnaire‐based asynchronous online tools and require real‐time consultations as part of service delivery [16, 17].

In this growing industry, there remains a knowledge gap in understanding why patients access these services, their health outcomes and how use of these services affects the broader primary care system in Australia, particularly general practice [2, 3]. Qualitative research on patient perspectives is important for informing decision‐makers who develop health policy and for gaining insight into the variety of patient values, experiences and needs regarding a specific health technology [18]. This study aimed to explore the perspectives and experiences of Australian patients using subscription‐based DTC telemedicine, focusing on benefits, concerns and implications for general practice. Through this research, we sought to enhance understanding of the therapeutic relationship between DTC telemedicine services and their patients, as well as the potential impacts of DTC telemedicine on traditional healthcare structures in Australia.

## 2 Materials and Methods

This qualitative research study involved semi‐structured interviews held remotely via Zoom. The methodological approach was grounded in post‐positivism, which recognises that while an objective truth exists, a person's comprehension of reality is inherently influenced by their experiences and perspectives [19]. This approach appreciates the significance of diverse viewpoints and the contextual factors that shape data interpretation. The conduct and reporting of this study followed the COnsolidated criteria for REporting Qualitative research (COREQ) checklist (see Table S1) [20].

### 2.1 Participants and Recruitment

Purposive sampling was used to recruit participants who had previously completed a survey on their experiences with up to five different DTC telemedicine services [21]. All services were owned by the same Australian digital healthcare company and generally focused on the following health areas: (1) weight loss, specifically marketed towards women; (2) sexual health and hair loss, specifically marketed towards men; (3) fertility and birth control, specifically marketed towards women; (4) skin care for both men and women, and (5) sexual well‐being for both men and women. In the survey, participants were asked if they would like to hear about future stages of research and potentially take part in an interview. Respondents who answered ‘yes’ were sent an email that described what the research was about, provided interview details and included a participant information and consent form. A sample size of 10–20 participants was targeted; this number was considered sufficient to enable data saturation, based on existing literature [22, 23].

Interviewees had no prior relationship with the researchers, and no participants dropped out or refused to participate after scheduling their interviews. Ethics approval was granted by the Human Research Ethics Committee at Macquarie University (Project ID: 23816), and all participants provided voluntary informed consent.

### 2.2 Data Collection

Interviews were conducted by A.T., a medical student completing this research as part of their Doctor of Medicine degree. Participants were aware of the interviewer's credentials, personal interest in the topic as a future health professional, and the research project's connection to a wider project investigating the emergence of digital healthcare technology in Australia [2, 3, 21, 24]. A.T. received training in qualitative interviewing and analysis from three research supervisors (L.A.E., K.C. and S.S.), all of whom are experienced in health services research and qualitative methods.

The interview guide incorporated open‐ended questions informed by both existing literature and the researcher's personal interests as a doctor in training with plans for a career in general practice (see Supporting Information 1) [2, 4, 5, 6, 7, 8, 9, 10, 11, 12, 13]. Questions were developed by A.T., refined through discussions with the research team and piloted with T.C.S., a fellow medical student with knowledge of the topic. Two members of the research team had experience using DTC telemedicine services, and their insights were drawn upon when developing the interview guide and later when interpreting findings. Participants had no prior relationship with A.T. and were asked about: their overall experiences using DTC telemedicine; previous and current general practice usage; and perceptions of the benefits of, and concerns with, DTC telemedicine services. The semi‐structured nature of the interview guide allowed for further prompting, or questions to be re‐ordered and clarified to promote elaboration on topics introduced by the participant [20]. Interviews were one‐off and lasted approximately 30–45 minutes. The sessions were recorded and automatically transcribed via Zoom. Transcripts were not reviewed by participants owing to time constraints.

### 2.3 Data Analysis

Data were analysed using Braun and Clarke's six phases of thematic analysis [25]. Transcripts were cleaned, and any identifying information was removed as part of the researchers familiarising themselves with the data. Transcripts were then imported into the NVivo 14 software for coding. Field notes were recorded by A.T. during and after interviews to facilitate drafting of the coding framework, which utilised both inductive (emerging patterns noticed during interviewing and reviewing transcripts) and deductive (from the literature) reasoning to generate initial codes. The draft framework had codes related to the relationship of DTC telemedicine with general practice; benefits of, and concerns with, DTC telemedicine; as well as double‐coding for the specific service that each quote was linked to. Revisions to the coding framework were made throughout the data analysis process to reflect developing themes and subthemes in the data (e.g., improved access to health advice, long general practice wait times and online interface became subcodes under the broader code of convenience and efficiency). A table of the coding tree with codes and subcodes, and including exemplar quotes, is provided in the Supporting Information (see Table S2). Codes and subcodes were then collated into overarching themes and subthemes. The developing themes were discussed with the research team during regular research meetings and refined iteratively to ensure the themes were conceptually distinct and that the interpretations accurately reflected the data.

## 3 Results

Thirteen participants were recruited (*n* = 11 female, *n* = 2 male; age range = 29–67 years; mean age = 47 years). Most participants lived in metropolitan areas (*n* = 10), with two participantsliving regionally, and one who lived rurally. All 13 participants had experience using at least one DTC telemedicine service, though two participants had discontinued their use of DTC telemedicine prior to the interview. Of these participants, seven engaged exclusively with the women's health service focused primarily on weight loss, three with the skin care service, two with the men's health service focused on sexual health and hair loss, and one with both the skin care service and women's fertility service. Following analysis of transcripts, four themes were identified that addressed our research questions: (1) convenience, efficiency and greater access; (2) the value of anonymity; (3) concerns about inappropriate use of medication; and (4) interactions with traditional general practice. The participant number is indicated in brackets after each verbatim quote.

### 3.1 Convenience, Efficiency and Greater Access

All participants highlighted the convenience of DTC telemedicine as a significant advantage, emphasising various aspects of the experience that improved upon traditional general practice. These aspects included flexible appointment scheduling, bypassing long general practice wait times, quick access to medications, availability of medical advice and the online user interface. For example, one participant with young children said:

The logistics that I as a mum of four have to go through to actually physically get myself to the GP, who inevitably is running late. And then… what was meant to be a 15 min appointment, really takes 1.5 to 2 hours out of my day. Having a telemedicine appointment that runs relatively on time, or at least you can get a text to say I'm a bit delayed, but I can take that call in the car, I can do whatever I'm doing, it's so convenient.

(P12)

Most participants indicated that DTC telemedicine could enhance access to medical advice not only for themselves but for populations with greater difficulty in physically presenting to healthcare services, such as rural and remote communities, the elderly and individuals with mobility challenges. For example:

We live in rural [state of Australia]. To get to my HRT [hormone replacement therapy] doctor, it's 75 kilometres each way. If I try and book with her, I have to book 3 or 4 months in advance to actually see her. I can send Juniper a message on the app, and I can talk to someone in 2 or 3 hours.

(P4)

Elderly people or people with disabilities that couldn't get out of the house as easily, that would certainly be an easy way of getting medical appointments.

(P10)

Despite a generalised perception among participants that DTC telemedicine could improve access to medical advice for those who lived far from healthcare services, some noted that there were still challenges with timely access to the medication provided through these online services. One participant residing in a rural area explained that her service's ‘delivery mechanism is coming out of [major city] and the company that they're using just never manages to actually deliver it on time’ (P4). Another participant acknowledged this issue, stating ‘I do know those in rural areas obviously experience those delays… I think I'm fortunate being in the CBD [central business district]’ (P9). While telemedicine was widely perceived as a more accessible and convenient alternative to traditional general practice, disparities in timely medication access, particularly in rural areas, highlight ongoing inequities in healthcare delivery.

### 3.2 The Value of Anonymity

DTC telemedicine services often address potentially stigmatised medical conditions such as obesity, erectile dysfunction and acne, offering a structured and condition‐specific approach to care. As one participant noted, ‘With specifically weight loss… people possibly don't want to go face‐to‐face to talk about their weight… because there is a bit of a stigma, even around the medication’ (P3). This quote reflects a broader preference common among participants for the semi‐anonymous nature of DTC telemedicine, which allowed individuals to seek care without the discomfort and potential judgement often experienced during in‐person consultations. Unlike traditional general practice, where patients often need to initiate uncomfortable discussions during a general consultation, DTC telemedicine services provide a streamlined, condition‐focused experience that typically begins with online screening tools rather than a face‐to‐face conversation. This approach was perceived as reducing psychological barriers to seeking care, encouraging some participants to engage with healthcare services they might otherwise avoid:

You're more likely to actually seek treatment because of that … Sometimes that conversation can be so incredibly difficult for people, and it can put them off going and getting the support that they need. And I think in situations like that, this kind of service would get more people willing to be open about what's going on and get help.

(P11)

Engagement with DTC telemedicine services for the treatment of potentially stigmatised medical conditions appeared to be driven by a preference for the structured, often asynchronous design, alongside targeted marketing and screening processes, which collectively help to mitigate stigma and empower patients to bypass traditional general practice pathways.

### 3.3 Concerns About Inappropriate Use of Medication

Despite recognition of the benefits of DTC telemedicine in providing patients with semi‐anonymity and greater control, more than half of participants expressed concerns that the services might inadvertently lead to inappropriate prescribing. For example, one participant stated, ‘You definitely have more control. Is it necessarily a good thing? I don't know. For me, I feel like I'm well informed. I feel like I have read all the things. I know a lot of people that haven't or won't’ (P12). Concerns were raised regarding the potential for circumvention of the traditional general practice gatekeeping system, with patients able to obtain medications despite ineligibility or prior denials from GPs. One participant stated, ‘Some people feel it is easier to access medication that they might not necessarily need, or … would not get if they saw a physical GP’ (P3). This consensus was most strongly felt by participants using the women's weight loss service who deemed that some patients accessed the service for aesthetic reasons rather than medical. One participant also raised the issue of inadequate patient education when using DTC telemedicine services, drawing on observations from online community forums:

It needs to be refrigerated, you need to know how to inject it, you need to know whether this is the right dose. And I've read, in the online forums, some really concerning stuff with people thinking they've got the wrong dose or, with the compound needles, misinterpreting that they should inject it all. So having 3 of the injections at once.

(P12)

Another participant highlighted the need for heightened regulation to prevent inappropriate prescribing and therefore ensure patient safety, explaining that ‘limitations on what can be prescribed would probably be worthwhile’ (P13).

### 3.4 Interactions With Traditional General Practice

Most participants sought DTC telemedicine services following negative experiences with GPs or pharmacists. Many expressed dissatisfaction with long wait times, exhaustion of treatment options, or insensitivity regarding their medical condition from traditional primary care providers, with one participant saying:

Because I felt like I had been quite open with the doctor, and I had gone to the dietician as they requested and done the things that they wanted me to do and hadn't seen the results. And so yeah, it… made me feel like rubbish, like I just felt worse, and I thought, I can't go to you again, and that is what pushed me in the direction of going to [the women's weight loss service].

(P8)

Participants' views on how DTC telemedicine impacted utilisation of general practice services were variable. Many suggested the frequency of GP visits was more closely tied to their satisfaction with prior GP encounters and the presence of other health conditions, given that general practice addresses a broader range of healthcare needs than the DTC telemedicine services they used. Whilst one participant said, ‘I see my GP regularly… I have osteoarthritis in my left knee, so I see him on a regular basis’ (P7), another explained, ‘I don't have any other ongoing… health conditions… I would only now go to the GP if I became like unexpectedly ill or something like that’ (P5), suggesting that, for patients without other health conditions, the convenience of DTC telemedicine may be sufficient to meet their healthcare needs.

Many participants reported an improvement in health outcomes from use of DTC telemedicine. As one participant explained, ‘Yes, I was using [the skin care service] for acne and I had a good experience with that. It was really easy, and it cleared up my acne really well’ (P5). However, participants frequently indicated a preference for general practice when it came to undifferentiated, or urgent medical presentations, where in‐person evaluation and a comprehensive approach are typically necessary:

Generally, if I need to go to the doctor it's something that's not over the phone. So like I found a lump under my arm, so I would go to a doctor, because they obviously have to feel it.

(P8)

The following quote further attests to how an established relationship with a GP was particularly important in the case of a chronic illness:

I guess I trust my GP, we've got a relationship. And because I have a complicated medical history, it means I don't have to start from scratch. She knows my medical history. She knows why I've been on the different medications I've been on… So I would think if something new was happening I'd rather go to my GP because I don't have to go through all that history.

(P6)

Although DTC telemedicine offered participants greater convenience and more specialised advice, these services lacked the rapport, trust, physical examination and holistic care experienced during face‐to‐face appointments with a GP.

I do think there's something to… be said for that face‐to‐face contact. But for the purposes of [my] skin… not really… If it was something more involved, I think I would want… to establish more of a rapport with the individual.

(P2)

DTC telemedicine services were also found to redirect patients back to their GP when clinical concerns arose that exceeded the scope of the service, as illustrated by the following participant account: ‘So when I talked to them about what was happening with my stomach… they said, “you probably need to go and talk to your GP”’ (P4). However, there was no integration of DTC telemedicine with general practice, as one participant noted, ‘I think my only concern is having to… relay the information from one source to another… They're not integrated’ (P9).

## 4 Discussion

This study highlighted convenience, efficiency and accessibility as major benefits of DTC telemedicine, with participants describing its flexibility and reduced wait times as improvements over traditional general practice. DTC telemedicine was described as facilitating care for stigmatised conditions (e.g., erectile dysfunction) by providing treatment pathways for patients who might be lost to follow‐up or never present to a GP in the first place. However, concerns were raised regarding patient safety, particularly in relation to inappropriate medication use. DTC telemedicine was also perceived as lacking the continuity of care, capacity for physical examination, holistic approach and patient‐provider trust that is inherent to traditional general practice, with participants primarily using DTC telemedicine for conditions for which they would not typically seek ongoing GP care. Despite this, negative experiences with GPs and pharmacists still appeared to encourage utilisation of DTC telemedicine.

Existing literature has similarly identified convenience as a major driver for DTC telemedicine use [8, 9, 11, 12, 21]. The potential of DTC telemedicine to bridge healthcare gaps for individuals living in rural areas or with mobility challenges has also been noted in previous studies [2, 7, 8, 11, 21]. However, disparities in delivery times between participants living in rural and metropolitan areas, combined with a lack of empirical evidence demonstrating that DTC telemedicine services adequately address this issue, suggest a need for further logistical improvements to ensure equitable access for all consumers, particularly those living outside of metropolitan areas [1]. Although concerns with cost and data safety have been observed in the literature, these were not a major issue among participants [11, 21]. Future research that incorporates the socioeconomic status of participants is essential to clarify variation in the financial accessibility of DTC telemedicine services, particularly in a country like Australia where patients often expect health services to be provided at little or no out‐of‐pocket cost.

Studies assessing the effect of DTC telemedicine on general practice utilisation remain limited, but one study found an increase in downstream use [13]. Our research suggests that the decision not to consult a GP was influenced by pre‐existing conditions and prior healthcare service experiences rather than the convenience of accessing DTC telemedicine alone. Participants reported that they generally used DTC telemedicine for concerns that they may not otherwise present to general practice for, such as obesity, skin care or erectile dysfunction. In contrast, GP services were preferred for management of chronic or complex conditions that require in‐person assessment and ongoing management. These findings suggest that DTC telemedicine services, particularly those focused on specific health conditions, are not replacing GP services but instead expanding access to care for individuals who might otherwise avoid general practice for these conditions. This finding aligns with a recent survey study of Australian users accessing DTC telemedicine services [21]. Fragmentation of care remains a concern, however, with a lack of integration between DTC telemedicine providers and traditional general practice leading to gaps in communication and continuity of care [5].

The implications of this study span across clinical practice, policy and future research directions. In the context of Australia's GP workforce shortages, with the projected undersupply worsening across all states and territories, and declining bulk‐billing rates increasing out‐of‐pocket costs for patients, the perceived convenience of DTC telemedicine is of particular significance [15]. The distinct strengths of both DTC telemedicine and general practice indicate a potential for collaboration within the primary care sector. DTC telemedicine in Australia typically caters to individuals with non‐urgent medical issues; therefore, its selective application may help reduce GP wait times for patients with time‐sensitive or more complex health needs. A focus on collaboration between DTC telemedicine providers and GPs could enhance efficiency, patient safety and continuity of care. Policies mandating the recording of DTC telemedicine consultations in My Health Record, Australia's national shared electronic health record, may improve interoperability and overcome concerns about care fragmentation. However, DTC telemedicine may also have unintended workforce implications if its growth encourages some GPs to pursue careers outside community primary care and towards DTC telemedicine services. Minimising disparities in medication delivery times between rural and metropolitan areas could position DTC telemedicine at the forefront of improving access to medical care for patients living in rural or regional areas. The development of robust quality assessment tools and comprehensive cost–benefit analyses is essential to ensure the effective integration of DTC telemedicine within Australian primary care [3]. Our findings indicate that negative experiences with general practice may influence some patients to explore DTC telemedicine, highlighting opportunities for GPs to address aspects of care that contribute to patient dissatisfaction. Potential strategies could include optimising appointment scheduling, improving clinical acumen and communication around stigmatised medical conditions and acknowledging DTC telemedicine options within traditional general practice models to provide a more seamless and patient‐centred approach to care. Importantly, while negative experiences with general practice, such as long wait times, may drive some patients towards DTC telemedicine services, these issues largely reflect systemic pressures within Australian primary care rather than shortcomings of individual GPs.

Although many participants reported positive health outcomes from DTC telemedicine use, the health impacts from the use of these services were not directly assessed by this study and remain an area for future research using longitudinal methods with larger samples. Concerns regarding inappropriate prescribing and inadequate patient education amongst users of the weight loss service are particularly pertinent to the current regulatory landscape given that DTC telemedicine services for weight loss frequently prescribe their users GLP‐1 receptor agonists [16]. Initiation of these medications requires individualised dose titration to mitigate the risk of gastrointestinal side effects and reduce the risk of more serious, albeit rare, adverse events, underscoring the significance of adequate patient education and monitoring [16, 26]. Therefore, greater oversight of DTC telemedicine prescribing practices is essential to ensure that patients fully understand their treatment regimen. Future research should investigate strategies to optimise this balance between accessibility, safety and appropriate prescribing to ensure favourable outcomes for patients using DTC telemedicine.

This is one of the few qualitative studies in the DTC telemedicine space that has explored patient perspectives on the use of DTC telemedicine, with findings related to the benefits, concerns and interactions between DTC telemedicine and traditional general practice. This paper offers critical insights for policymakers, GPs and researchers that may not be evident from quantitative studies, and that are directly applicable to healthcare delivery in Australia [3, 18]. This study was conducted on participants recruited through a prior survey study published by members of the same research group [21]. The present study extends the qualitative component of the survey study, which consisted of two open‐response survey questions. Semi‐structured interviews enabled a rich and in‐depth exploration of participant experiences, revealing the social contexts underpinning patterns observed in quantitative data, while generating hypotheses for future research.

Several limitations of this study should be acknowledged. The sample size was small (*n* = 13), with participants recruited entirely from users of services operated by a single Australian digital healthcare company. Furthermore, 7 of the 13 participants used the women's weight loss service, which may have impacted on the types of concerns and benefits raised (e.g., related to stigma, prescribing safety) and may not be characteristic of users of other types of services, such as more generalised DTC telemedicine. Participants were recruited from those who agreed to be contacted regarding future research after completion of a prior survey, which introduced selection bias and may have shaped a more favourable view of DTC telemedicine within the themes.

## 5 Conclusion

This qualitative study explored the perspectives of 13 users of DTC telemedicine, focusing on the perceived benefits and concerns with the model of care, as well as its influence on general practice utilisation. Consistent with existing literature, convenience and accessibility were identified as key benefits, particularly for those facing barriers to in‐person care or those seeking treatment for stigmatised conditions. Primary concerns centred on loose prescribing practices which appeared to lead to inappropriate use of medications by some users, and the absence of continuity of care when compared to traditional general practice. While participants primarily used DTC telemedicine for conditions they may not otherwise visit a GP for, they frequently compared DTC telemedicine favourably with traditional services. The benefits and concerns identified in this study, in combination with the increasing popularity of DTC telemedicine, underscore the need for a structured approach when integrating services into the Australian healthcare system. By addressing regulatory gaps, preventing care fragmentation through collaboration with general practice and ensuring equitable access across different patient populations, DTC telemedicine may complement and ultimately strengthen the primary healthcare system in Australia.

## Author Contributions

**Andrew Turner:** methodology, writing – original draft, writing – review and editing, conceptualization, formal analysis. **Kate Churruca:** conceptualization, writing – review and editing, methodology, supervision. **Samantha Spanos:** methodology, supervision, writing – review and editing. **Thomas Courtney‐Stubbs:** writing – review and editing, methodology. **Darran Foo:** writing – review and editing, conceptualization. **Louise A. Ellis:** conceptualization, methodology, supervision, writing – review and editing.

## Funding

The authors have nothing to report.

## Ethics Statement

Ethics approval was granted by the Human Research Ethics Committee at Macquarie University (Re: 520241256855842), and all participants provided voluntary informed consent.

## Conflicts of Interest

The authors declare no conflicts of interest.

## Supporting information


Supporting File

## Data Availability

The data that support the findings of this study are available on request from the corresponding author. The data are not publicly available due to privacy or ethical restrictions.
