# Supplementary material for: Use of Direct‐to‐Consumer Telemedicine: A Qualitative Study Exploring the Thoughts, Perspectives and Experiences of Australian Adults
Source: Health Expect. 2026 Aug 31;29(5):e70847. doi: 10.1111/hex.70847 (PMC13527857; doi:10.1111/hex.70847)
Supplement: Supplementary file 1 [file HEX-29-e70847-s001.docx]

**SUPPLEMENTAL MATERIAL**

**Supplemental Table 1: COREQ (COnsolidated Criteria for REporting Qualitative research) Checklist**

| **Topic** | **Item No.** | **Guide Questions/Description** | **Reported on Page No.** |
| --- | --- | --- | --- |
| **Domain 1: Research team and reflexivity** | | | |
| *Personal characteristics* | | | |
| Interviewer/facilitator | 1 | Which author/s conducted the interview or focus group? | AT |
| Credentials | 2 | What were the researcher's credentials? E.g. PhD, MD | AT, MD student |
| Occupation | 3 | What was their occupation at the time of the study? | AT, MD student |
| Gender | 4 | Was the researcher male or female? | AT (Male) |
| Experience and training | 5 | What experience or training did the researcher have? | AT received training in qualitative interviewing and analysis from research supervisors (KC, LAE, SS) (page 6) |
| *Relationship with participants* | | | |
| Relationship established | 6 | Was a relationship established prior to study commencement? | The interviewer had no prior relationship with the interviewees (page 6) |
| Participant knowledge of the interviewer | 7 | What did the participants know about the researcher? e.g. personal goals, reasons for doing the research | Participants were informed about the study aims before agreeing to participate. Participants were aware that the research was conducted as part of the interviewer’s Doctor of Medicine degree. (page 6) |
| Interviewer characteristics | 8 | What characteristics were reported about the interviewer/facilitator? e.g. Bias, assumptions, reasons and interests in the research topic | AT is a medical student (page 6) |
| **Domain 2: Study design** | | | |
| *Theoretical framework* | | | |
| Methodological orientation and Theory | 9 | What methodological orientation was stated to underpin the study? E.g. grounded theory, discourse analysis, ethnography, phenomenology, content analysis | Qualitative study, grounded in post-positivism (page 5) |
| *Participant selection* | | | |
| Sampling | 10 | How were participants selected? E.g. purposive, convenience, consecutive, snowball | Survey participants who expressed interest in taking part in an interview (page 5-6) |
| Method of approach | 11 | How were participants approached? E.g. face-to-face, telephone, mail, email | Email (page 5-6) |
| Sample size | 12 | How many participants were in the study? | 13 (page 7) |
| Non-participation | 13 | How many people refused to participate or dropped out? Reasons? | No participants dropped out or refused to participate after scheduling their interviews (page 6) |
| *Setting* | | | |
| Setting of data collection | 14 | Where was the data collected? E.g. home, clinic, workplace | Zoom (page 5) |
| Presence of non-participants | 15 | Was anyone else present besides the participants and researchers? | N/A |
| Description of sample | 16 | What are the important characteristics of the sample? E.g. demographic data, date | Demographic data was not collected |
| *Data collection* | | | |
| Interview guide | 17 | Were questions, prompts, guides provided by the authors? Was it pilot tested? | A semi-structured interview schedule was developed (pages 6-7) |
| Repeat interviews | 18 | Were repeat interviews carried out? If yes, how many? | Single interview conducted (page 7) |
| Audio/visual recording | 19 | Did the research use audio or visual recording to collect the data? | Audio recorded (page 7) |
| Field notes | 20 | Were field notes made during and/or after the interview or focus group? | Field notes were made during and after interviews (page 7) |
| Duration | 21 | What was the duration of the interviews or focus group? | Interviews lasted approximately 30-45 minutes (page 7) |
| Data saturation | 22 | Was data saturation discussed? | A sample size of 10-20 participants was considered optimal for data saturation to be reached based on existing literature (page 6) |
| Transcripts returned | 23 | Were transcripts returned to participants for comment and/or correction? | Transcripts were not returned to participants for comment or correction (page 7) |
| **Domain 3: analysis and findings** | | | |
| *Data analysis* | | | |
| Number of data coders | 24 | How many data coders coded the data? | AT (page 7) |
| Description of the coding tree | 25 | Did authors provide a description of the coding tree? | Description provided in Supplemental table 2 |
| Derivation of themes | 26 | Were themes identified in advance or derived from the data? | A combination of inductive and deductive analysis was conducted to identify themes (page 7) |
| Software | 27 | What software, if applicable, was used to manage the data? | NVivo (page 7) |
| Participant checking | 28 | Did participants provide feedback on the findings? | Participants did not provide feedback on the findings |
| *Reporting* | | | |
| Quotations presented | 29 | Were participant quotations presented to illustrate the themes/findings? Was each quotation identified? e.g. participant number | Participant quotations are provided, with participant number provided in brackets (pages 8-13) |
| Data and findings consistent | 30 | Was there consistency between the data presented and the findings? | Quotations are presented to illustrate key findings (pages 8-13) |
| Clarity of major themes | 31 | Were major themes clearly presented in the findings? | Yes key findings are presented (pages 8-13) |
| Clarity of minor themes | 32 | Is there a description of diverse cases or discussion of minor themes? | Minor themes are discussed (pages 8-17) |

**Supplemental Table 2: Coding framework**

|  | **Codes** | **Subcodes** | **Participant Quote** |
| --- | --- | --- | --- |
| **Benefits** | Convenience and efficiency (n=13) | Long wait times for GP (n=11) | “I had a GP that I did really like, and I tried to book in with them, but it's so hard to book in with them like you have to book 2 to 3 weeks in advance to see them, and generally. I A: I don't have my roster that far ahead of time, and B: I don't know that I need to see a doctor that far ahead of time.” - P8 |
|  |  | Busy work/family life (n=8) | “Yeah, I've always gone to my GP to get the script for the contraceptive pill, but I have 3 small children. So just getting out of the house with them, and I would have to take them to appointments with me, and it's just hard to have a conversation with an adult when I've got them there.” - P5 |
|  |  | Improved access to health services: rural and remote communities, mobility issues, elderly (n=10) | “So you know we live in rural New South Wales. To get to my HRT doctor, it's 75 kilometres each way. If I try and book with her, I have to book 3 or 4 months in advance to actually see her. I can send Juniper a message on the app, and I can talk to someone in 2 or 3 h. They always say you know, it could be 24 to 48 h, but generally, if you've got a medical problem and it sounds bad, they'll be on to you immediately, whereas I can ring up my doctor locally here and say, I've got this problem, and they can say "we can see you in 9 weeks".” - P4 |
|  |  | Access to medication (n=12) | “Okay. So I started using this primarily because I couldn't get the medication. I went to my GP and she suggested that I try Saxenda. Then I found I couldn't purchase it, so I saw Juniper advertising that they could supply it.” - P4 |
|  |  | Online interface (n=11) | “I find it really easy to use. It's a really good interface. Really manageable, makes it much more efficient, quick, you can clearly understand it. I've I actually really like the layout of it and just the fact that you don't need to go and see someone, it's all online. It makes it just very convenient.” - P2 |
|  | Cost (n=6) |  | “For example, I pay 134 bucks for 8 weeks. I'm pretty sure the equivalent from chemist warehouse was 150 something so, and that's posted to me direct to home.” - P13 |
|  | Anonymity and stigma (n=11) |  | “Then I suppose with specifically weight loss, it is the anonymity as well that you know, people possibly don't want to go face to face, to talk about their weight and getting medication for it, because there is a bit of a stigma, even like to the medication.” - P3 |
|  | Greater autonomy (n=8) |  | “Yep, I definitely do, you definitely have more control. Is it necessarily a good thing? I don't know. For me. I feel like I'm well informed. I feel like I have read all the things. I know a lot of people that haven't or won't.” - P12 |
|  | Improved health outcomes (n=6) |  | “In the time that I've been with them, I've lost something like 25 kilos I think so far so. And through the platform and what they've been doing in terms of the fitness. I'm exercising 6 days a week now. I'm doing a lot more things. I'm a lot more active. I'm a lot more confident. My weight, which I'd struggled with for probably, not for lack of trying, but I'd struggled with for probably I reckon 10 years. So I've had a really a really positive experience with them and a lot of support from them through that process and 100%. It's achieving what I set out for it to achieve, which was to be healthier.” - P11 |
|  | Specialised (n=5) |  | “It's more like, I think that the doctors that are doing the software are probably, you know, they have more knowledge on the skin issues than my general practitioner does. You know half of the general practitioners I see they don't know half as much as I do about this stuff.” - P2 |
| **Concerns** | Medication quality (n=6) |  | “Yeah, I found when it went to the compound medication. It didn't seem to be as effective as the brand name medication. So I'm just persevering and seeing how that goes.” - P6 |
|  | Prescribing Practices  (n=9) | Inappropriate prescribing (n=8) | “But also, I suppose there's the other side of things, which maybe is a concern, that some people feel it is easier to access medication that they might not necessarily need, or sort of would not get if they saw a physical GP, so there's that that element.” - P3 |
|  |  | Inadequate instructions on dosage (n=1) | “And I've read in the online forums, I've read some really concerning stuff with people thinking they've got the wrong dose or with the compound needles, misinterpreting that they should inject it all. So, having 3 of the injections at once, like, Oh, I've just taken 3 needles because I looked at this. The mls and the dosage, like they're different, like a syringe will be 0.37 mls and it will say it's a 0.5 dose. So people think that that's not enough. So they take 2 injections like this is not okay. It's not okay. And then they go and post it on the online forum for all of us to see and everyone else corrects them. And I'm thinking this is not clearly spelt out enough. This is unsafe.” - P12 |
|  |  | Support with side effects (n=3) | “I wouldn't be bothered going to them for it, like I'll just, if I had an adverse event, I'd be off to my doctor, and you know I'd stop taking the medication because it'd probably be related to that. I'd go see my GP. And then talk to them about it, so I wouldn't bother.” - P13 |
|  | Interface difficulty/technology (n=7) |  | “I think there's a generation of people that would rather do things online than face to face whereas I would imagine it's too hard for a lot of people, I mean even I in the beginning I was a bit confused with the app, like where to get information which information was held where. You know it takes a little while to work it out.” - P6 |
|  | Medication delivery (n=6) | Not on time (n=6) | “But then again, shortages hit, and their delivery system hasn't been working properly. And so sometimes I've ended up missing 3 doses before I've finally got my dose.” - P4 |
|  |  | Refrigeration (n=3) | “Not everyone has jobs where you can go. Oh, I'll just duck home quickly and pick that because you get a text when it arrives. But you can't just leave work and take it and put in the fridge. And then I had to. I was like, I can't be home for 5 h, and it's 35 degrees. So you just paid a stupid amount of money for medication.” - P8 |
|  | Data safety and personal information (n=2) |  | “I didn't like doing it, but if you think about it, we do it all the time. With Facebook, or you know you sign up to a mailing list for anything; you've already given them your name, address, phone number, email, address. I didn't like doing it, and you hope that they have good security. You know, stuff to look after your data.” -P8 |
|  | Cost (n=8) |  | “Not just a little bit, like I'd be happy to pay a little bit more and I was happy initially. Then, when I found out what the labelled cost is for the official product. $130 versus $375. You're charging someone 3 times. That's really a lot. To me, an alarming concern.” - P12 |
|  | Communication (n= 12) | Lack of face-to-face interaction and physical exam (n=11) | “I think it would depend on the scenario. I think there are certain things where people need to touch and feel and physically see. and there are others where you might be able to submit blood tests, and whatever. It would really depend on the situation, like if I was, if I had suspicious lumps, or you know, moles or bits. Like there's certain things that you would, that need to be eyeballed. And there's other things. I don't need to hop on the scales in front of the doctor. Do you get what I mean?” - P11 |
|  |  | No continued relationship (n=6) | “I'd mostly be more comfortable for sort of bigger things to have that one person. I know that there is a doctor that I talk to whenever I have, like they would be my doctor. I'd be open to have teleconsultations with them, but I'd sort of like to know that they have my history. They know who I am, and then we talk to, I talk to them about my medical concerns.” - P3 |
| **Interaction with General Practice** | Effect on GP visits (n=11) | No effect (n=5) | “I mean, outside of you know, not having that initial appointment with him about it. It hasn't changed how many times I've have had to have been to a doctor, and I haven't sort of not been to a doctor because of it. I jumped on this before I actually had a doctor. So yeah, not really.” - P13 |
|  |  | Increase in visits (n=2) | “I think it did. I think it made me, rather than sit at home doing nothing, I think it's inspired me just to keep persisting with the GPs like. Okay, well, I've tried that option, and that option hasn't worked for me. So since coming off Juniper, I've had a heap more GP appointments, if that's kind of what you're asking and I've just been more persistent.” - P12 |
|  |  | Decrease in visits (n=5) | “I don't feel as though that I need to attend the GP as regularly as what I would used to. I think a lot of why I was attending previously. I like, I guess I'm using my juniper, and my health has seen a drastic like improvement, so I don't feel as though that I need to go as regularly to my GP.” - P9 |
|  | Negative experiences with doctors and pharmacists in the past (n=9) |  | “Yeah, I would say, past experiences with going to the GP and having to book in and having to like attend an actual consultation hasn't been overly positive, whereas this way it was just I didn't have to go through that whole process. Yes, so it was a lot more positive in that aspect.” - P9 |
|  | Degree of comparability to General Practice (n=13) |  | “But I, I think they're 2 very different things for me, anyway. Very different thing. Yeah, because I think, yeah, as a GP… you've got a wide array of things that you need to deal with. But these services are very much specialised on the specific area.” - P8 |
|  | Existing collaboration between GP and telemedicine (n=6) | Yes (n=4) | “I would go and see my GP and even perhaps get his opinion and say, Look, I'm thinking of doing that, that that in the future. What do you think and see if he was comfortable about that as well, so we could work almost in conjunction with in, you know, in person, visits and telemedicine use of telemedicine. I don't see that they have to be mutually exclusive.” - P7 |
|  |  | No (n=2) | “I guess my only concern is if something, I guess did happen, like if I had some major side effects, and I do have to go see my GP, like if it's a 10 out of 10, I am not getting any better. I don't feel as though they are helping like. If I have to get to that point. I think my only concern is having to, I guess, relay the information from one, I guess source to another source and having to go through. I guess the medical history with Juniper back to my GP, like it's there's obviously not. They're not integrated.” - P9 |

**Supplemental material 1: Semi-structured interview guide**

Thank you for taking part in this quick interview. My name is (researcher), and I am a medical student who is completing this research as part of a wider project at the (research institute) that looks at the emergence of digital health care technology in Australia. It might have been some time since you participated in the original survey, which asked about your use of direct-to-consumer telemedicine services. This conversation is an extension of that survey. Throughout this interview I will use the word telemedicine to refer to subscription services like (the 5 DTC telemedicine services studied). When we talk about telemedicine in this interview, we are not talking about services such as telehealth consultations with doctors, online videos, or free educational websites. What I am interested in are the reasons that Australians use telemedicine as well as their effect on health outcomes and primary care utilisation. Today I will be asking you a series of questions about this and recording your responses for research purposes only. Is that ok?

Did you have any questions before we get underway?

Current/prior experience with telemedicine?

- What telemedicine services have you used?
- Can you tell me about your experience using this service/s so far?
- Were you sceptical before use and why?
- Did the use of telemedicine lead to the desired health outcome? Why/why not?
- What personal information have you provided to this telemedicine service throughout your interaction?

Primary care

- Did you try to manage your medical condition via traditional medical consultations first?
- Have you talked to a GP about using telemedicine? Why/why not?

If yes:

A. How did they respond?

B. Do you use this resource collaboratively with your primary care provider

C. Do you think these discussions influenced the outcomes that occurred?

D. Has that affected your usage of digital healthcare technologies since?

- Has your use of telemedicine influenced your experiences and the number of interactions with a GP? Why/why not?
- How does telemedicine compare with the care you get from a GP?

Benefits and concerns

- What are some benefits that you found of using telemedicine as opposed to face-to-face consultations with a GP?
- Do you have any concerns about using telemedicine. If so, could you explain what they are?
- What do you think are some of the reasons that other people might choose to seek out and use telemedicine for their health?

1. Cost
2. Greater autonomy `
3. Improving health literacy
4. unhelpful consultations with doctors in past/second opinion
5. access to medications not available through traditional primary care/second opinion
6. Easy access/availability of physical appointments/remote nature
7. Anonymity/stigma surrounding medical condition
8. Urgency of medical condition

- What do you think are some of the reasons why other people might choose not to use telemedicine?

1. Interface difficulty
2. Confidentiality issue/privacy issues
3. Data safety
4. Quality of service/effectiveness of product
5. Cost/upselling/feeling overcharged
6. Availability of medications
